# Comparison of local infiltration analgesia and interscalene block for postoperative pain management in shoulder arthroscopy: a prospective randomized controlled trial

**DOI:** 10.3906/sag-2008-57

**Published:** 2021-06-28

**Authors:** Olgun BİNGÖL, Alper DEVECİ, Semih BAŞKAN, Güzelali ÖZDEMİR, Enver KILIÇ, Emrah ARSLANTAŞ

**Affiliations:** 1 Department of Orthopedics and Traumatology, Ankara City Hospital, Ankara Turkey; 2 Department of Orthopedics and Traumatology, Private Ortadogu Hospital, Ankara Turkey; 3 Department of Anesthesiology, Ankara City Hospital, Ankara Turkey; 4 Department of Orthopedics and Traumatology, Sinop Boyabat 75. Yıl Hospital, Sinop Turkey

**Keywords:** Functional scores, interscalene block, local infiltration analgesia, postoperative pain management, rotator cuff repair, shoulder arthroscopy

## Abstract

**Background/aim:**

The aim of this study was to compare the effects of local infiltration analgesia and interscalene brachial plexus block techniques on postoperative pain control and shoulder functional scores in patients undergoing arthroscopic rotator cuff repair.

**Materials and methods:**

Sixty patients who underwent arthroscopic rotator cuff repair were prospectively included in the study. Patients were randomly divided into two groups. Group 1 was comprised of patients who had interscalene brachial plexus block, while group 2 was comprised of patients who had local infiltration analgesia. In group 1, interscalene block was applied with 20 mL 0.5% bupivacaine. In group 2, the Ranawat cocktail was used for local infiltration analgesia. Sixty milliliters of Ranawat cocktail was applied to the subacromial space and glenohumeral joint in equal amounts. Postoperative pain was assessed by the VAS score. Functional scores of the shoulder were also evaluated by Constant–Murley and UCLA scores. The time of first analgesic requirement and total analgesic consumption in the postoperative period were assessed.

**Results:**

The first analgesic requirement was significantly late in the interscalene brachial plexus block group (p = 0.000). There was no statistically significant difference between the groups in terms of total analgesic consumption (p = 0.204). In the postoperative 6th h, the VAS score was 2.43 in the interscalene brachial plexus block group, whereas 2.86 in the local infiltration analgesia group (p = 0.323). There was no statistically significant difference between the groups in terms of Constant–Murley shoulder and UCLA scores in the 3rd postoperative month (respectively, p = 0.929, p = 0.671). Besides, postoperative VAS scores and functional scores were negatively correlated (p < 0.01).

**Conclusion:**

Local infiltration analgesia is an effective alternative to interscalene brachial plexus block for postoperative pain management and total analgesic consumption in arthroscopic rotator cuff repair. However, the interscalene brachial plexus block provides a longer postoperative painless period.

## 1. Introduction

Among the orthopedic complaints, shoulder pain ranks third following knee pain and low back pain [1]. Arthroscopy procedure for the treatment of shoulder joint diseases is a frequently used treatment method [2].

Most patients who underwent shoulder arthroscopy describe pain in the postoperative period [3]. Approximately 75% of these patients classify pain severity as moderate, severe, or extreme [4]. Patients who do not have adequate pain control in the postoperative period may experience decreased quality of life, loss of function, increased complications, and permanent postoperative pain.

Treatment success in shoulder arthroscopy depends on good rehabilitation in the early period. The efficiency of postoperative rehabilitation depends on the effective application of multimodal analgesia methods [5]. Therefore, many multimodal analgesia methods are mentioned in the current literature. Interscalene brachial plexus block (IBPB) has been reported to provide effective analgesia [6,7]. The content of a local infiltration anesthetic, which is reported to be as effective as an interscalene block in shoulder arthroscopy, has not been disclosed to the best of our knowledge [3,8,9]. Ranawat cocktail is one of the local infiltration analgesia methods used for effective postoperative pain management in knee and hip surgery [10,11]. Ranawat cocktail was used for local infiltration analgesia in the shoulder joint for the first time in our study.

The aim of this study was to compare the effects of local infiltration analgesia and IBPB techniques on postoperative pain control and postoperative functional scores in patients undergoing arthroscopic rotator cuff repair.

Our null hypothesis was that there was no significant difference between the local infiltration analgesia and IBPB in terms of postoperative pain management in a patient undergoing arthroscopic rotator cuff repair.

## 2. Materials and methods

This prospective, randomized study was conducted in a single-center after the approval by Ankara Numune Training and Research Hospital Ethical Committee (E-18-1910; approval date: 08/05/2018). All the researchers who participated in the study signed the most recent version of the Helsinki Declaration. The informed consent form was obtained from the patients in the study.

G*Power 3.1.9.4 program was used for power analysis. Sample size was calculated using the VAS score as the primary effect variable. Power analysis was calculated by determining 0.89 effect value and 0.95 power ratio for the VAS score. The sample size was calculated as a total of 56 patients, 28 patients for both groups. In order to increase the power of the study, we determined the sample size of the study as 60 patients.

Between May 2018 to March 2019, 60 patients with American Society of Anesthesiologists (ASA) physical state I-II-III and rotator cuff repair with shoulder arthroscopy were included in the study. Only patients with double row suture anchor configuration for the arthroscopic treatment of full-thickness rotator cuff tears were included in the study.

Patients with ASA physical status IV-V, infection at the site of application, local anesthetic allergy, other shoulder pathologies, acromioclavicular impingement, bleeding diathesis, and cervical disc problems were excluded from the study. Patients who may have vocal cord paralysis, respiratory failure, neuromuscular disease, and neuropathy were also excluded from the study. Patients with massive and partial rotator cuff tears were excluded from the study.

The patients in the study were randomized according to the sequentially numbered, sealed opaque envelope method. The patients included in the study were randomly divided into two groups. Group 1 was comprised of patients who had IBPB, while group 2 was comprised of patients who had local infiltration analgesia. General anesthesia was applied to all patients by the same author/anesthesiologist. 

### 2.1. Interscalene brachial plexus block technique (IBPB)

All nerve blocks were made postoperatively by the same author/anesthesiologist. After the block area was sterilized using povidone-iodine, the nerve was observed using a 5.0–13.0 MHz linear probe (LOGIQ e; GE Healthcare, Princeton, NJ, USA). After the transducer was placed in the transverse plane, the carotid artery at the level of the cricoid cartilage was detected. The brachial plexus between the interscalene muscle groups was identified by directing the transducer laterally across the neck. A 21G needle (Techniplex, 50 mm, 30°; Vygon, Ecouen, France) was advanced until it was inserted between the C5 and C6 nerve trunks. After careful aspiration to prevent intravascular injection, the block was applied with 20 mL 0.5% bupivacaine [8].

### 2.2. Local infiltration analgesia (LIA)

In group 2, the Ranawat cocktail was used for local infiltration analgesia in our study. Sixty milliliters of Ranawat cocktail was applied to the subacromial space and glenohumeral joint in equal amounts by the surgical team at the end of the surgical procedure. The analgesic solution used in our study was designed by taking the cocktail sample applied by Ranawat Orthopedic Center after knee and hip surgery [10]. The content of the analgesic solution used in our study is shown in Table 1.

**Table 1:** Local anesthetic solution content.

Medication	Strength/dose	Amount
Bupivacaine	200 mg	40 cc
Epinephrine	0.15 mg	0.3 cc
Dexamethasone	8 mg	2 cc
Cefuroxime	750 mg	7.5 cc
Sodium chloride	0.9%	10.2 cc

All arthroscopic surgery procedures in the study were performed by the same senior author orthopedist. Shoulder arthroscopy was performed using the shoulder table in a beach-chair position. Arthroscopic rotator cuff repair was performed in all patients included in the study, and the double row technique was applied. At the end of the operation, no drain was placed in the surgical area and operation time was recorded.

### 2.3. Postoperative follow-up

Sex, age, operation side, and ASA score of the patients were collected. Moreover, postoperative complications and side effects were recorded.

Patients included in the study were followed closely for 24 h postoperatively for local anesthetic toxicity. Lipid emulsion treatment was planned when a serious side effect was observed indicating local anesthetic toxicity. Moreover, appropriate airway management equipment was made available for necessary situations. In addition, cardiac monitoring was planned for patients with toxic effects.

The patients included in the study were informed in advance for pain monitoring and their consent was obtained for 24-h follow-ups at the hospital. Patients were checked at 0, 1, 6, and 24 h and their pain status were questioned with a VAS scale from 0 to 10. To ensure standardization in treatment, tramadol 100 mg iv infusion was started in both groups to the patients who needed it as an additional analgesic method (VAS ³ 4). Tramadol administration time was recorded as the first analgesic requirement time. Total analgesic consumption was also collected. 

The VAS scale was required and evaluated for the operated shoulder at the postoperative 3rd week, 6th week, and 3rd month follow-up. In addition, shoulder functions of the patients were evaluated with Constant–Murley and The University of California-Los Angeles (UCLA) scores at 6 weeks and 3 months postoperatively.

### 2.4. Statistical evaluation

Statistical analysis was performed using SPSS 22.0 (IBM Corp., Armonk, NY) for Windows. The Shapiro–Wilk “Sig” values ​​were examined to see whether the series showed normal distribution. The Mann–Whitney *U* test was performed to determine the statistically significant difference between parameters not showing normal distribution. Chi-square test was used for the comparison of the categorical variables such as sex, ASA score, side, and analgesic consumption. The results were evaluated in 95% confidence interval and p < 0.05 was considered significant. Since the data were not normally distributed, the correlation analysis of the postoperative VAS score with the early functional scores was performed with Spearman’s RHO test.

## 3. Results

A total of 60 patients were included in the current study. The mean age of all patients was 52.05 ± 12.7 years. The patients consisted of 32 males and 28 females. Arthroscopic rotator cuff repair was performed in all patients included in the study. The right side of 44 patients and the left side of 16 patients were operated. In addition, the mean value of the ASA score in all patients was 2 ± 0.36 (Table 2).

**Table 2:** Demographic data.

	IBPB (n = 30)	LIA(n = 30)	Total(n = 60)	p
Age (years)	54.4 ± 9.9	49.6 ± 14.7	52.05 ± 12.7	0.143
Sex (male/female)	17 / 13	15 / 15	32 / 28	0.605
Side (right/left)	24 / 6	20 /10	44 / 16	0.243
ASA score	2.03 ± 0.32	1.97 ± 0.41	2 ± 0.36	0.488

The demographic data of the patients included in the study according to the groups are shown in Table 2. There were no statistically significant differences between groups in terms of age, sex, side, and ASA score (respectively: p = 0.143, p = 0.605, p = 0.243, p = 0.488) (Table 2).

Evaluations of first analgesic requirement time and total analgesic consumption are summarized in Table 3. The first analgesic requirement time was significantly late in the IBPB group (p = 0.000). When the total analgesic consumption of the patients was compared, there was no statistically significant difference between the groups (p = 0.204).

**Table 3:** Evaluation of first analgesic requirement time, and total analgesic consumption between groups.

	IBPB	LIA	p
First analgesic requirement time (hour)	15.96 ± 1.70	7.86 ± 1.65	0.000
Total analgesic consumption	1.0 ± 0.24	1.1 ± 0.84	0.204

The values are presented as mean ± SD.

There was no significant difference between the groups in terms of postoperative 6th h VAS scores (Figure) (p = 0.323). In addition, 3rd week, 6th week, and 3rd month postoperative VAS scores did not show significant difference between the groups (respectively, p = 0.913, p = 0.503, p = 0.28) (Figure). 

**Figure:**
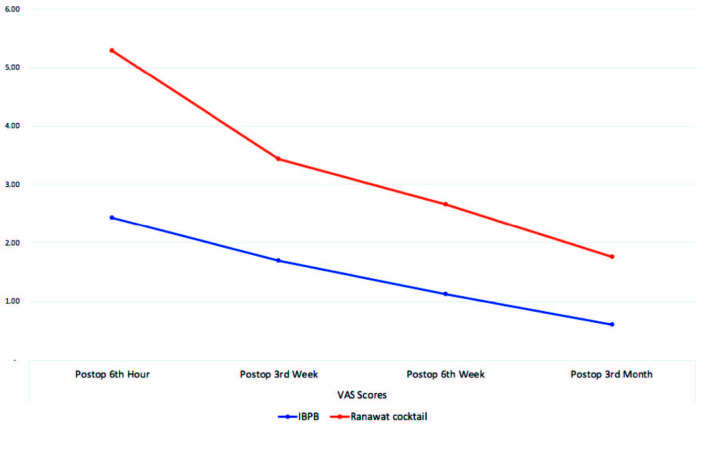
Comparison of postoperative VAS scores depending on the analgesic at surgery.

Sixth week and 3rd month postoperative Constant–Murley shoulder scores were similar in both groups (respectively, p = 0.929, p = 0.318) (Table 4). Moreover, there was no significant difference in the postoperative sixth week and third month UCLA scores of the two groups (respectively, p = 0.776, p = 0.671) (Table 5). 

**Table 4:** Comparison of Constant–Murley score between groups.

		IBPB	LIA	p
Constant–Murley score	Postop 6th week	33.1 ± 2.4	34.8 ± 1.95	0.318
	Postop 3rd month	75.2 ± 2.09	74.9 ± 2.35	0.929

The values are presented as mean ± SD.

**Table 5:** Comparison of UCLA between groups.

		IBPB	LIA	p
UCLA	Postop 6th week	19.1 ± 0.70	19.03 ± 0.80	0.776
	Postop 3rd month	28.4 ± 0.59	27.6 ± 0.97	0.671

The values are presented as mean ± SD.

Correlation analysis of postoperative 6th h VAS score and postoperative Constant–Murley shoulder (r = 0.392, p = 0.002) and UCLA scores (r = 0.433, p = 0.001) showed a statistically significant negative correlation.

Besides, in all patients in the two groups included in our study, no complications or side effects related to the analgesic method applied were observed.

## 4. Discussion

The most important finding of this study was that there was no statistically significant difference between the local infiltration analgesia and the IBPB in the postoperative VAS scores and the total analgesic consumption. 

Most patients who underwent shoulder arthroscopy describe pain in the postoperative period [3]. When effective pain control is not performed in the postoperative period, patients have both susceptibilities to chronic pain and retardation in functional recovery [5]. Multimodal analgesia is reported to be an effective method in postoperative pain management in previous studies [5]. For these reasons, we used multimodal analgesia methods in our study.

IBPB is frequently used for postoperative pain control in shoulder arthroscopy [12]. Although IBPB provides effective analgesia, it has many defined side effects. Permanent nerve injury, respiratory failure, pneumothorax, and cardiovascular complications are among the main side effects [13]. In addition, the effectiveness of IBPB is influenced by technical difficulties and anesthesiologist-dependent conditions. In the current study, there were no side effects and complications related to the IBPB.

Local infiltration analgesia is a simple, safe, and effective analgesic technique [14]. In the literature, there are many studies about local infiltration analgesia injection into the shoulder joint [3,8,9,15,16]. However, no studies have been found in the literature regarding the use of Ranawat cocktail in the shoulder joint. Since the other local analgesia solutions mentioned in the literature after shoulder arthroscopy were not effective enough in postoperative pain management, The Ranawat cocktail which was successfully used in the knee joint [10,11,17,18] was applied into the shoulder joint. Although no vascular complications have been reported in local infiltration analgesia applied to the shoulder joint, vascular complications may be observed. It is recommended by the authors to apply local infiltration analgesia carefully during injection. In this study, no complications were observed in patients who underwent the LIA.

Corticosteroids cause time- and dose-dependent effects on the articular cartilage. The beneficial effects can be observed at low doses and shorter durations, whereas detrimental effects can be seen at high doses and longer durations [19]. In our study, the prepared Ranawat cocktail was divided in half and equal amounts of solution were injected into both the subacromial space and glenohumeral joint. Thus, it was aimed to apply low-dose steroid injection to the articular spaces in the study. Moreover, no adverse effect related to the articular cartilage was observed in the study patients.

Dietz et al. reported that the volume of the glenohumeral joint of 60 patients was approximately 43 mL [20]. Yi et al. noted that the volume of the subacromial space was 20–30 mL [21]. Matziolis et al. found that the knee joint volume in their study was 131 ± 53 mL [22]. In our study, since the volumes of the knee and shoulder joints were similar, we applied the amount of cocktail used by the Ranawat Orthopedic Center on the knee to the shoulder joint.

Iliaens et al. reported that IBPB is the gold standard for pain control in shoulder surgery but offers effective pain control up to 8 h postoperatively [23]. In our study, patients who underwent IBPB needed additional analgesics after 15.96 h. The first analgesic requirement time was significantly late in the IBPB group.

Laurila et al. reported that IBPB effectively reduced early postoperative pain and opioid need compared to subacromial bursa block after shoulder arthroscopy [3]. Baskan et al. noted that continuous interscalene block reduces postoperative pain more safely and effectively in the open shoulder surgery [8]. Souvatzoglou et al. reported that continuous interscalene nerve blockade was a better postoperative analgesia control compared to continuous subacromial administration of ropivacaine in shoulder surgery [9]. Bjørnholdt et al. noticed that the local infiltration analgesia (150 mL ropivacaine 0.2% and 0.25 mg epinephrine) technique cannot be recommended for postoperative pain management after shoulder replacement unless substantially modified [16]. Webb et al. found that there was no significant difference between continuous subacromial infusion versus interscalene block with postoperative pain scores [15]. In another study, it was reported that interscalene block and subacromial bursa block had similar effects in postoperative pain management [14]. In the literature, comparative studies of local infiltration analgesia and IBPB concluded that they had a similar effect on pain control, or that IBPB was more effective. In the current study, the authors selected Ranawat cocktail with proven efficacy in the knee joint for local infiltration analgesia. Moreover, the authors applied Ranawat cocktail to both subacromial space and glenohumeral joint. It is known that the most common pain generator in the shoulder joint is the biceps root, the bicipital groove, and the subacromial region [24]. Biceps, biceps labral complex, and capsular pathologies accompany both chronic subacromial and cuff disorders [24]. Hence, applying local infiltration analgesia only to the subacromial area is not enough for effective pain control, which was also observed in our clinical experiences. Therefore, we tried to affect the regions with dominant pain generators in the shoulder joint by applying the local infiltration analgesia both subacromially and in-capsule.

Beiranvand et al. reported that liposomal bupivacaine had better postoperative analgesic control compared with the injected bupivacaine usage [25]. A metaanalysis showed that liposomal bupivacaine was as effective as interscalene nerve block in reducing pain scores after shoulder arthroplasty [26]. In the current study, injection of Ranawat cocktail which contains bupivacaine was compared with interscalene block. However, there is a need for future researches comparing the effects of Ranawat cocktail and liposomal bupivacaine usage in the shoulder arthroscopy.

In this study, we found that local infiltration analgesia is an effective alternative to interscalene block for postoperative pain management and total analgesic consumption in shoulder arthroscopy. Besides, there was no statistically significant difference found in terms of Constant–Murley and UCLA scores in postoperative 6th week and 3rd month. In addition, the administration of analgesic solution to the glenohumeral joint positively affects the postoperative pain management of the patient.

The limitation of our study is the use of data performed in a single-center, on a single-shoulder pathology, and a small number of patients. The strength of our study is that Ranawat cocktail was used for the first time in the shoulder joint for local infiltration anesthesia. Moreover, Ranawat cocktail and interscalene block for postoperative pain management in shoulder arthroscopy were compared for the first time in the literature in our study. In addition, being a randomized controlled study increases the value of our study.

The most important advantage of local infiltration analgesia is that it can be easily performed by orthopedic surgeons when the anesthesiologist does not have sufficient experience in the IBPB, or the equipment is not available for the block.

## 5. Conclusion

Local infiltration analgesia is an effective alternative to IBPB for postoperative pain management and total analgesic consumption in shoulder arthroscopy. However, the IBPB provides a longer postoperative painless period. Local infiltration analgesia can be applied for postoperative pain management in patients with IBPB contraindicated.

## Disclaimers

There is no funding source for this study. No benefits in any form have been received or will be received from a commercial party related directly or indirectly to the subject of this article. The authors received no financial support for the researches, authorship, and/or publication of this article. 

## References

[ref1] (2002). Rotator cuff disease. Current Orthopaedics.

[ref2] (2020). Arthroscopic rotator cuff repair using a transosseous knotless anchor (ATOK). Journal of Shoulder and Elbow Surgery.

[ref3] (2002). Interscalene brachial plexus block is superior to subacromial bursa block after arthroscopic shoulder surgery. Acta anaesthesiologica Scandinavica.

[ref4] (2016). Management of postoperative pain: a clinical practice guideline from the american pain society, the american society of regional anesthesia and pain medicine, and the American Society of Anesthesiologists’ Committee on Regional Anesthesia, Executive Commi. The Journal of Pain.

[ref5] (2010). Postoperative analgesia for shoulder surgery: a critical appraisal and review of current techniques. Anaesthesia.

[ref6] (2019). A comparison of continuous interscalene block versus general anesthesia alone on the functional outcomes of the patients undergoing arthroscopic rotator cuff repair. European Journal of Orthopaedic Surgery & Traumatology.

[ref7] (2019). Patient-reported outcomes after arthroscopic shoulder surgery with interscalene brachial plexus nerve block using liposomal bupivacaine: a prospective observational study. Surgical Technology International.

[ref8] (2017). Comparison of continuous interscalene block and subacromial infusion of local anesthetic for postoperative analgesia after open shoulder surgery. Journal of Orthopaedic Surgery.

[ref9] (2007). Continuous interscalene nerve blockade versus continuous subacromial administration of ropivacaine for postoperative pain management in shoulder surgery. Regional Anesthesia & Pain Medicine.

[ref10] (2009). Multimodal pain management after total hip and knee arthroplasty at the Ranawat Orthopaedic Center. Clinical Orthopaedics and Related Research.

[ref11] (2019). Post-operative pain management using local infiltration analgesia (LIA) in total knee arthroplasty (TKA): A prospective study. International Journal of Orthopaedics Sciences.

[ref12] (2018). Interscalene brachial plexus bolus block versus patient-controlled interscalene indwelling catheter analgesia for the first 48 hours after arthroscopic rotator cuff repair. Journal of Shoulder and Elbow Surgery.

[ref13] (2007). The types and severity of complications associated with interscalene brachial plexus block anesthesia: local and national evidence. Journal of Shoulder and Elbow Surgery.

[ref14] (2008). Subacromial bursa block is an effective alternative to interscalene block for postoperative pain control after arthroscopic subacromial decompression: a randomized trial. Journal of Shoulder and Elbow Surgery.

[ref15] (2007). Continuous infusion of a local anesthetic versus interscalene block for postoperative pain control after arthroscopic shoulder surgery. Arthroscopy : the journal of arthroscopic & related surgery.

[ref16] (2015). Local infiltration analgesia versus continuous interscalene brachial plexus block for shoulder replacement pain: a randomized clinical trial. European Journal of Orthopaedic Surgery & Traumatology.

[ref17] (2009). Efficacy of intra-articular cocktail analgesic injection in total knee arthroplasty—a randomized controlled trial. The Knee.

[ref18] (2011). Effect and safety of periarticular high-dose bupivacaine injections during total knee arthroplasy in patients with heart diseases. Orthopedic Journal of China.

[ref19] (i5). Utility of arthroscopic guided synovial biopsy in understanding synovial tissue pathology in health and disease states. World journal of orthopedics 2014 Nov; 5.

[ref20] Intra-articular volume assessment in glenohumeral instability. Knee surgery, sports traumatology.

[ref21] (2015). Subacromial volume and rotator cuff tears: Does an association exist. Indian Journal of Orthopaedics.

[ref22] (2015). Wagner A. The volume of the human knee joint. Archives of Orthopaedic and Trauma Surgery.

[ref23] (2019). Regional anaesthesia for surgical repair of proximal humerus fractures: a systematic review and critical appraisal. Archives of Orthopaedic and Trauma Surgery.

[ref24] (2000). The incidence of pathologic changes of the long head of the biceps tendon. Journal of Shoulder and Elbow Surgery.

[ref25] (2018). Bupivacaine versus liposomal bupivacaine for pain control. Drug Research.

[ref26] (2017). Liposomal bupivacaine versus interscalene nerve block for pain control after shoulder arthroplasty: A meta-analysis. Medicine (Baltimore).

